# Evolving Epidemiology and Emerging Antifungal Resistance in Vulvovaginal Candidosis: Evidence from a Five-Year Survey

**DOI:** 10.3390/pathogens15050538

**Published:** 2026-05-16

**Authors:** Aristotelis Tsiakalos, Despoina Papageorgiou, Vassiliki C. Pitiriga, Christina Vogiatzi, Panayotis D. Ziakas, Ioannis Routsias, Evangelia Dimitroulia, Karolina Akinosoglou

**Affiliations:** 1 “Hygeia” General Hospital, 6 Erythrou Stavrou Street, 15123 Marousi, Greece; atsiakalos@gmail.com; 2 Department of Internal Medicine, University General Hospital of Patras, 26504 Rio, Greece; dspn.pap96@gmail.com; 3 Department of Medicine, University of Patras, 26504 Rio, Greece; 4 Department of Microbiology, Medical School of Athens, National and Kapodistrian University of Athens, 75 Mikras Asias Str., 11527 Athens, Greece; siliapit@hotmail.com (V.C.P.); jroutsias@yahoo.com (I.R.); evidim@med.uoa.gr (E.D.); 5 “Leto” General, Maternity & Gynecology Clinic, Mouson 7-13 Street, 11524 Athens, Greece; cvogiatzi@leto.gr; 6 Department of Medicine, Brown University, Providence, RI 02912, USA; pd.ziakas@gmail.com; 7 Division of Infectious Diseases, University General Hospital of Patras, 26504 Rio, Greece

**Keywords:** *Candida albicans*, non-*albicans Candida*, vaginosis, vulvovaginal candidosis

## Abstract

*Candida* species, particularly *Candida albicans* (*C. albicans*), are the leading cause of vulvovaginal candidosis (VVC) among women of reproductive age. In recent years, the epidemiology of VVC has shifted toward non-*albicans Candida* (NAC) species, accompanied by increasing antifungal resistance. This retrospective study evaluated the epidemiological profile of VVC and antifungal susceptibility patterns in Greece between 2020 and 2024 at a tertiary maternity and gynecological hospital. Species identification was performed using the VITEK^®^ 2 system, and antifungal susceptibility followed EUCAST guidelines. A total of 526 vaginal swab samples were analyzed, comprising *C. albicans* (57.9%) and NAC species (42.1%). The median age was 36.3 years (range: 18–92). Among NAC isolates, *Nakaseomyces glabratus* (26.4%) predominated, followed by *Pichia kudriavzevii* (7.6%), *Candida parapsilosis* (5.1%), and *Candida tropicalis* (3.0%). Resistance rates among *C. albicans* isolates increased from 9.3% in 2020 to 22.3% (fluconazole) and 20.9% (itraconazole) in 2024. Voriconazole resistance was not detected until 2023 and 2024, when rates rose to 3.2% and 15.2%. Fluconazole resistance was observed in *C. parapsilosis* (3.5%) and *C. tropicalis* (12.5%). Echinocandin resistance remained low overall, except for *N. glabratus*, which demonstrated a 13.8% resistance rate to caspofungin. These findings highlight the need for surveillance, improved diagnostics and antifungal stewardship.

## 1. Introduction

Vulvovaginal candidosis (VVC) is a common condition, accounting for approximately one-third of all cases of vulvovaginitis among women of reproductive age. It is estimated that 70–75% of women will experience at least one episode during their lifetime, while 5–8% will develop recurrent infection, defined as four or more episodes per year [1]. Previous estimates suggest that the prevalence of recurrent VVC will increase substantially by 2030, with nearly 158 million new cases occurring annually [2]. *Candida albicans* (*C. albicans*), a common commensal of the genitourinary tract, has historically been the predominant species isolated from clinical samples of women diagnosed with VVC. However, non-*albicans Candida* (NAC) species have recently attracted increasing scientific and epidemiological interest. NAC prevalence has risen globally, accounting for approximately 10–45% of VVC cases in some studies [3,4,5]. Interestingly, Dan et al. found a higher rate of NAC species in asymptomatic women (44.5%) compared to those with sporadic (19.4%) or chronic (21%) vaginitis [6].

The diagnosis of VVC remains challenging and often relies on clinical suspicion, which may lead to inappropriate treatment. Even though gold standard diagnostic methods include wet-mount microscopy and microbiological culture, they are time-consuming and may be underutilized in routine clinical practice due to limited physician training [7,8,9]. Of note, in the United States, over 60% of VVC cases lack diagnostic testing, potentially leading to misdiagnosis and inappropriate treatment [10], underlining the pressing need for rapid and accurate diagnostic tests [11]. Similarly, treatment approaches also differ significantly among healthcare providers. While fluconazole is commonly prescribed for VVC [10,12], prescription patterns vary based on physicians’ training backgrounds [12]. The availability of over-the-counter (OTC) antifungal medications since 1991 has further influenced prescribing trends [13]. OTC antifungal medications for VVC are cost-effective for uncomplicated cases but carry significant risks of misdiagnosis and inappropriate use for other gynecologic conditions. Previous evidence has shown that only 34.5% of those with a prior VVC diagnosis could correctly diagnose VVC from a classic case scenario [14]. More problematically, 14.6% of previously diagnosed women would inappropriately use OTC antifungals for bacterial vaginosis compared to 6.4% of medically trained controls. OTC preparations are the least costly to the healthcare system for infrequent acute VVC, provided the diagnosis is correct [15]. However, OTC use risks delaying diagnosis of potentially serious sexually transmitted diseases like *Chlamydia* or *Trichomonas* that present with similar symptoms [16]. The evidence suggests OTC antifungals benefit low-risk patients with confirmed prior VVC diagnoses but pose diagnostic risks in first-time users [14,17,18].

As a result of antifungal misuse, the emergence of antifungal resistance poses an additional challenge to the management of fungal infections. A substantial number of studies indicate a rise in fluconazole-resistant *C. albicans* strains [19,20]. In addition, the inherent azole resistance, as well as the acquired resistance mechanisms of NAC species have become a growing issue, as these species are increasingly being identified in cases of VVC and treatment options remain limited [21]. Even though, no significant correlation between OTC azole exposure and drug-resistant *Candida* colonization has been confirmed, resistant isolates from women with multiple OTC exposures represent an emerging concern following years of indiscriminate drug prescription and unnecessary drug exposure [22,23].

Nonetheless, precise data on the frequency of VVC are limited, as the condition is not a reportable disease and is frequently self-diagnosed without clinical or laboratory confirmation. Among symptomatic women with microbiologically confirmed infection, VVC prevalence varies widely by country and population. Reported prevalence rates range from 5.3% to 60%, with higher rates observed in countries such as Tunisia, Nigeria, Australia, and Brazil [24,25,26,27,28]. Data from Greece on the epidemiology and antifungal susceptibility of *Candida* species causing VVC remain limited, with the most recent evidence derived from studies by Maraki et al. exploring trends between 2012 and 2017, and by Kroustali et al. assessing epidemiological patterns over a two-year period [28,29,30]. This lack of up-to-date national data, along with the reported global variability in VVC epidemiology, highlight the need for contemporary surveillance of species distribution and antifungal resistance patterns. In this context, we aimed to investigate the epidemiology of VVC caused by *C. albicans* and NAC species by analyzing their prevalence and distribution over a five-year period (2020–2024), thereby providing updated insights into recent epidemiological trends. In addition, we evaluated the antifungal susceptibility profiles of *Candida* species for five different antifungal agents, including fluconazole, itraconazole, amphotericin B, caspofungin and anidulafungin providing a summary of antifungal resistance rates over a five-year period.

## 2. Materials and Methods

### 2.1. Study Design and Patient Enrolment

We conducted a retrospective descriptive analysis of data obtained from laboratory records of female patients who tested positive for symptomatic VVC caused by *C. albicans* or NAC species at Leto General, Maternity and Gynecology Clinic S.A., Athens, Greece, between 2020 and 2024. The study population consisted of consecutive women presenting with symptoms of VVC. Inclusion criteria were women aged 18 years or older presenting with symptoms consistent with VVC. Diagnosis was based on clinical symptoms and confirmed by microbiological culture. Only the first isolate per patient was included in the analysis to avoid duplication. Patients with missing file data or without microbiological confirmation were excluded from this analysis.

The study was conducted in accordance with the Declaration of Helsinki and the principles of Good Clinical Practice and was approved by the relevant ethics committee and the local institutional review board (approval no. 394/28.12.2023, 395/28.12.2023). The data collection and analysis started in August 2024 and was completed by the end of the year. The retrospective nature of the study was determined by the fact that all data and samples had already been collected as part of standard clinical practice prior to their inclusion in the research analysis.

### 2.2. Sample Collection and Microbiology Methods

We retrospectively analyzed clinical and laboratory records of women who underwent vaginal swab sampling for routine diagnostic purposes between 2020 and 2024. Vaginal swabs were collected aseptically from the vaginal fornix and vaginal wall and transported promptly to the microbiology laboratory under controlled conditions. Samples were cultured on Sabouraud dextrose agar with chloramphenicol (Bio-prepare, Keratea, Greece) and incubated aerobically at 35–37 °C for 24–48 h. Positive cultures were identified by macroscopic and microscopic examination.

Species identification and antifungal susceptibility testing were performed using the VITEK^®^ 2 automated system (bioMérieux, Marcy l’Étoile, France) with AST-YS cards. Isolates were subcultured on Sabouraud dextrose agar at 35 ± 2 °C for 24 h, and yeast suspensions were prepared in sterile 0.45% saline adjusted to a 0.5 McFarland standard [31], using a DensiCHEK Plus densitometer (bioMérieux, Marcy l’Étoile, France). AST-YS cards, containing predefined antifungal concentrations in RPMI-1640-based medium, were incubated automatically at 35 ± 1 °C, with turbidity measured every 15 min for up to 48 h. *Candida parapsilosis* ATCC 22019 and *Pichia kudriavzevii* ATCC 6258 were used as quality-control strains. Minimum inhibitory concentrations (MICs) were interpreted according to EUCAST clinical breakpoints version 11.0 (valid from December 2024), and isolates were categorized as susceptible (S), intermediate (I), or resistant (R). Previous validation studies have demonstrated >95% concordance between AST-YS and reference broth microdilution methods [32].

### 2.3. Statistical Analysis

Data were analyzed using descriptive statistical methods. Results were presented as frequencies and percentages for each fungal species to assess annual epidemiological trends, as well as the distribution of pathogens across different age groups. Inferential statistics, including chi-square tests, were applied to evaluate differences in species distribution across age groups. Statistical significance was set at *p* < 0.05. Annual resistance rates for each antifungal agent were expressed as percentages per year. Trends in antifungal resistance over time were assessed using the Cochran–Armitage Trend Test. Statistical analyses were performed using SPSS software, version 29.0.2.0.

## 3. Results

### 3.1. Study Population and Species Distribution

A total of 526 cases of VVC were identified between January 2020 and June 2024. The mean age of the participants was 36.28 years (range: 18–92 years). *C. albicans* was the most prevalent species, accounting for 57.9% of cases, followed by *Nakaseomyces glabratus* (*N. glabratus*) (26.4%), *P. kudriavzevii* (7.6%), *C. parapsilosis* (5.1%), and *Candida tropicalis* (*C. tropicalis*) (3.0%). No mixed infections involving two or more fungal species were detected. The distribution of *Candida* isolates during the study period is presented in Table 1.

### 3.2. Age-Specific Distribution of Pathogens

A statistically significant difference in pathogen distribution was observed across age groups (*p* < 0.001) (Supplemental Table S1). Among women aged 50 years and older, NAC species were identified in 59.6% of cases, whereas *C. albicans* accounted for 40.5%.

**Figure 1:**
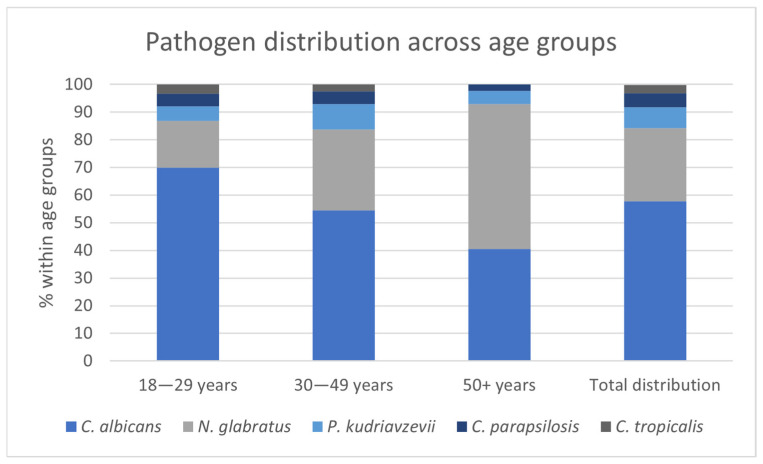
Pathogen distribution across age groups.

In contrast, *C. albicans* vaginitis predominated in younger women, occurring in 69.9% of those aged 18–29 years and 54.5% of women aged 30–49 years. NAC species were detected in 30.1% and 45.5% of women in these respective age groups. The age-related distribution of pathogens is illustrated in Figure 1.

### 3.3. Antifungal Susceptibility of C. albicans

Antifungal susceptibility testing results for *C. albicans* isolates over the study period are summarized in Table 2. Resistance to fluconazole and itraconazole increased from 9.3% in 2020 to 22.3% and 20.9% in 2024, respectively. The highest resistance rates were observed in 2022, reaching 23.8% for fluconazole and 31.0% for itraconazole. Voriconazole resistance increased from 3.2% in 2023 to 8.7% in 2024. No resistance to amphotericin B or caspofungin was detected until 2023, with resistance rates remaining low at approximately 0.8% in 2024. No significant trends in antifungal resistance were observed over the study period.

### 3.4. Antifungal Resistance Among NAC Species

Resistance rates among NAC species are summarized in Table 3. Owing to relatively small annual sample sizes, cumulative resistance rates for the entire study period are reported. *N. glabratus* and *P. kudriavzevii* demonstrated intrinsic resistance to azole antifungals. In contrast, *C. tropicalis* and *C. parapsilosis* exhibited resistance to fluconazole, with overall resistance rates of 12.5% and 3.7%, respectively. Resistance to amphotericin B among *N. glabratus* and *P. kudriavzevii* was 13.7% and 13.2%, respectively. Overall, amphotericin B resistance among NAC species increased from 10.9% in 2020, peaked at 17.9% in 2022, and subsequently declined to 5.9% in 2024. Echinocandin resistance remained low throughout the study period; however, *N. glabratus* exhibited a resistance rate of 13.8% to caspofungin. Among resistant isolates, 7.8% demonstrated resistance to two or more antifungal agents. NAC species demonstrated statistically significant trends in resistance over the study period, particularly for fluconazole (*p* = 0.006). Consistent with NAC findings, a statistically significant trend in fluconazole resistance over time was identified when all species were analyzed together (*p* < 0.01).

## 4. Discussion

VVC is a common fungal infection associated with substantial morbidity and a significant negative impact on women’s quality of life. This study investigated the epidemiology and antifungal susceptibility trends of VVC in Greece over a five-year period (2020–2024). In our cohort, *C. albicans* (57.9%) was the predominant species, followed by NAC species (42.1%). NAC species became increasingly prevalent with advancing age. We observed rising resistance rates among both *C. albicans* and NAC species across multiple antifungal regimens over time.

Our findings support the emerging shift in the microbial epidemiology of VVC toward NAC species and are consistent with reports from other regions, including a study from Ethiopia that documented comparable species distribution patterns [33]. Higher occurrence of NAC vaginitis was found in studies conducted in India and Lebanon [34,35], while lower incidence was observed in China, Kuwait and Brazil [36,37,38]. In southern Europe, earlier and more recent studies conducted in Italy consistently reported *C. albicans* as the predominant species, followed by *N. glabratus* [39,40]. Prior investigations in Greece reported *C. albicans* as the most frequently isolated species followed by *C. parapsilosis* in one study [29], and *N. glabratus* in another [28]. Comparable findings with *N. glabratus* as the second most prevalent species have also been observed in studies from Turkey [41]. No published data were identified from other countries in the region, including Cyprus, Albania or Spain.

*N. glabratus* accounted for more than half of NAC-associated VVC cases in our study, in line with previous reports [28]. Most studies identify *N. glabratus* as the predominant NAC species, representing approximately 50–66% of NAC-related vaginitis. However, the distribution of NAC species among women with VVC varies by geographic region and population. Accordingly, the second most prevalent NAC species differ across studies, with *C. tropicalis*, *C. parapsilosis*, and *P. kudriavzevii* reported at varying frequencies [3,6,36]. Notably, pathogen distribution varied significantly across age groups in our study, with NAC species being more frequently isolated in women aged 50 and above. These results were consistent with previous studies, which reported higher prevalence of NAC species in older women with VVC [28,40,42]. Prior exposure to antifungal agents and the use of hormone replacement treatment may be associated with this observation [3]. Shifting epidemiology may partly be attributed to the introduction of OTC antifungals and short-course treatments, which effectively suppress *C. albicans* but may allow overgrowth of other species [3,43]. On top of that, age-related changes in vaginal microbiota and immune response may favor colonization by NAC species [1].

In the present study, no cases of mixed *Candida* infections were identified among the samples analyzed. This may reflect methodological limitations, as culture techniques may underestimate mixed infections due to overgrowth of a single species. Previous studies from Greece have reported a low prevalence of mixed infections [28], whereas more recent investigations in Greece and other countries have demonstrated substantially higher rates, reaching up to 8% in Greece and 29.3% in other regions. Mixed *Candida* infections represent an additional challenge in the management of VVC [29,44].

For *C. albicans* the resistance rates for fluconazole and itraconazole increased from 9.3% in 2020 to 22.3% and 19.0% in 2024 respectively. Voriconazole resistance rose from 3.2% to 8.7% during 2023 and 2024. The observed resistance rates are significantly higher than those reported by previous studies conducted in Greece, in which a 4.3% and a 6.6% overall resistance to fluconazole was reported [28,29]. Similarly, lower resistance rates have been reported in other regions including China, the United Kingdom, Vietnam, and Iran [45,46,47,48]. The findings regarding azole resistance raise significant concerns, especially due to indiscriminate and unnecessary fluconazole exposure evoking a number of resistance mechanisms [23]. Although resistance mechanisms were not investigated in the present study, previous reports have associated the overexpression of efflux pump genes (*CDR1*, *CDR2*, and *MDR1*) to azole resistance in some isolates [49]. In addition, *ERG11* gene mutations and overexpression are more prevalent in fluconazole-resistant strains [50]. Clonal spread of specific genotypes, particularly CC69 and DST79, is associated with fluconazole non-susceptibility in VVC patients [50]. Resistance mechanisms are not limited to *C. albicans*; similar patterns have been observed in *N. glabratus*. Among NAC species, *N. glabratus* and *P. kudriavzevii* exhibit intrinsic azole resistance. In contrast, *C. parapsilosis* and *C. tropicalis* showed resistance to fluconazole with an overall rate of 3.7% and 12.5% respectively. Other than efflux pumps, *Candida* species can develop resistance to various antifungal classes through alterations in drug targets and biosynthetic pathways [51].

Regarding echinocandin resistance, both *C. albicans* and NAC species demonstrated high susceptibility rates throughout the study period, and our findings are consistent with data reported in previous years in Greece [28,30]. However, echinocandin resistance in *Candida* species, particularly *N. glabratus*, has emerged as a growing concern in treating VVC and invasive candidiasis [52,53]. In the present study, *N. glabratus* exhibited a resistance rate of 13.8% to caspofungin. Resistance mechanisms involve mutations in *FKS* genes encoding glucan synthase, the target enzyme of echinocandins, thus reducing drug sensitivity and increasing minimum inhibitory concentrations [53]. Echinocandin resistance typically develops after 3–4 weeks of treatment and is associated with poor outcomes [54]. Standardized susceptibility testing methods can detect resistant strains, but variability exists among clinical laboratories [55].

The overall resistance rate to amphotericin B for NAC species increased reaching a peak of 17.9% in 2022, before decreasing to 5.9% in 2024. A recent meta-analysis of 63 studies reported wide variability in amphotericin B resistance rates for *C. parapsilosis* ranging from 0% to 46.9%, with an overall pooled rate of 1.3% [56]. For cases involving azole-resistant *Candida* species, vaginal suppositories containing amphotericin B have been used successfully. In a previous study, 99.8% of *C. albicans* isolates and all NAC isolates (100%) were found to be susceptible to amphotericin [28].

The management of VVC is based on international treatment guidelines and recommendations. For uncomplicated cases most organizations recommend either a single-dose oral fluconazole regimen or a short course of a topical azole regimen such as clotrimazole or miconazole. However, the convenience of a single-dose treatment makes fluconazole the preferred option for many women [57,58,59,60,61,62,63,64,65]. Fluconazole is also recommended as maintenance treatment in recurrent VVC cases often in combination with longer courses of topical therapies [57,58,61,62,63,65]. To this end, the widespread use of fluconazole has undoubtedly contributed to the emergence of azole-resistant *Candida* strains. Authors have previously suggested the use of a more tailored-driven approach especially in cases of recurrent VVC, e.g., ReCiDiF (Recurrent *Candida* infections treated with degressive individualized doses of fluconazole) scheme in order to curb increasing resistance rates and rationalize use of systemic antifungals [66]. At the moment, for NAC vaginitis, intravaginally suppositories of boric acid or nystatin are preferred [57,60,61,62]. In Greece, VVC management generally follows the international recommendations. Topical treatments, including amphotericin B and miconazole/econazole vaginal suppositories are also available. However, data on relative frequency of use of specific antifungal treatments is lacking partly because these agents are available OTC.

Currently, the use of new echinocandins including caspofungin, anidulafungin and micafungin, is evaluated for their efficacy in treating resistant infections. Caspofungin has demonstrated promising results in treating mucosal and invasive candidiasis that is unresponsive to other therapies, with response rates ranging from 82% to 100% [67,68]. Data is limited on micafungin or anidulafungin efficacy on VVC, except for isolated reports as rescue therapy [69], necessitating more clinical trial before their use in this setting. However, echinocandins are currently only available for intravenous administration are not approved for the treatment of superficial infections such as VVC.

Despite the available treatment options, the shifting epidemiology of *Candida* species and growing antifungal resistance have driven the exploration of novel therapies [70]. Ibrexafungerp belongs to a newer class of antifungals called triterpenoids, inhibiting β-(1,3)-D-glucan synthesis, an essential component of the fungal cell wall, similar to echinocandins, however providing the option of oral administration. Ibrexafungerp has demonstrated in vitro activity comparable or superior to echinocandins, including against resistant *N. glabratus* strains [71]. Oteseconazole, on the other hand, represents a next-generation azole, inhibiting fungal CYP51 lanosterol demethylase; thus, blocking ergosterol synthesis. Its key advantage lies on the much higher selectivity for fungal enzymes vs. human CYP enzymes, hence resulting in fewer drug–drug interactions than classic azoles and reduced toxicity profile. Oteseconazole showed strong activity against azole-resistant *C. albicans* and NAC species in the treatment of chronic recurrent VVC [72]. Moreover, adjunctive treatments including probiotics [73], as well as, the ongoing development of an immunotherapeutic vaccine (NDV-3A)—currently in clinical trials—offers the potential to revolutionize the prevention of VVC [21,74]. However, the long-term success of these innovations will rely heavily on their judicious use, guided by informed clinical decision-making and robust antifungal stewardship to ensure sustainable and effective outcomes [21,75,76].

This study has several important limitations, including its retrospective observational single-center design. Although a large number of samples were analyzed, detailed clinical information was unavailable, including prior exposure to antifungal or antibiotic therapy, use of hormone replacement therapy, pregnancy status, underlying comorbidities and treatment history, limiting the ability to assess clinical risk factors and treatment outcomes. Further limitations relate to the diagnostic approach used, which did not include wet-mount microscopy. Therefore, differentiation between true VVC and vaginal colonization may have been limited. In addition, while all included cases involved symptomatic patients, the analysis did not allow differentiation between recurrent and non-recurrent episodes. The biofilm-forming capacity of *Candida* isolates from recurrent VVC patients is heterogeneous and associated with altered antifungal sensitivity, particularly to fluconazole [77]. Interestingly, *C. albicans* can switch phenotypes during successive episodes of recurrent VVC, potentially contributing to treatment challenges [78]. Moreover, in a number of cases, insufficient evidence regarding established clinical breakpoints for certain fungal species precluded in vitro susceptibility assessment. Additionally, limitations associated with the use of automated susceptibility testing include variability compared to reference broth microdilution methods and occasional misidentification of certain NAC species. It should also be noted that, even when minimum inhibitory concentrations are determined, fluconazole resistance identified through in vitro susceptibility testing does not always correlate with clinical (phenotypic) resistance. The phenomenon of trailing growth exhibited by *Candida*—a result of its diverse resistance mechanisms and ability to form biofilms—can obscure the true relationship between in vitro susceptibility and in vivo treatment efficacy [79,80]. Additionally, negative culture results do not necessarily rule out active infection; they may instead reflect recent antifungal treatment, poor-quality swab collection, the presence of inhibitory substances, or sampling errors that miss the site of infection. Various types of rapid molecular diagnostic tests for VVC have become broadly available with promising performance characteristics; however, their use complicates antifungal susceptibility testing [11,81].

To conclude, our findings demonstrate a substantial burden of NAC species and an alarming increase in azole resistance among VVC isolates, underscoring the evolving therapeutic challenges in contemporary practice. The emergence of resistance even among traditionally susceptible species highlights the urgent need for continuous local epidemiological surveillance, accurate species-level identification, and routine antifungal susceptibility testing to guide targeted therapy and avoid inappropriate empirical or OTC antifungal use. Strengthening antifungal stewardship programs, increasing clinician awareness regarding resistance trends and NAC pathogens, and promoting rational antifungal prescribing are essential to limit further resistance emergence and preserve antifungal efficacy. Future multicenter studies integrating clinical, microbiological, and molecular resistance data are warranted to better inform treatment strategies and public health interventions.

## Figures and Tables

**Table 1:** *Candida* sp. distribution during the study period.

*Candida* sp.	2020 *n* (%)	2021 *n* (%)	2022 *n* (%)	2023 *n* (%)	2024 *n* (%)	2020–2024 *n* (%)
*C. albicans*	54 (45.8)	26 (27.4)	42 (51.9)	61 (79.2)	121 (78.1)	304 (57.9)
*N. glabratus*	46 (39.0)	41 (43.2)	23 (28.4)	4 (5.2)	25 (16.1)	139 (26.4)
*P. kudriavzevii*	12 (10.2)	13 (13.7)	8 (9.9)	3 (3.9)	4 (2.6)	40 (7.6)
*C. parapsilosis*	6 (5.1)	9 (9.5)	4 (4.9)	4 (5.2)	4 (2.6)	27 (5.1)
*C. tropicalis*	0	6 (6.3)	4 (4.9)	5 (6.5)	1 (0.6)	16 (3.0)
Total cases/year	118	95	81	77	155	526

Abbreviations: *C. albicans*: *Candida albicans*, *N. glabratus*: *Nakaseomyces glabratus*, *P. kudriavzevii*: *Pichia kudriavzevii*, *C. parapsilosis*: *Candida parapsilosis*, and *C. tropicalis*: *Candida tropicalis*.

**Table 2:** *C. albicans* resistance rates for tested antifungal agents.

Antifungal Agent	2020 *n* (% Resistance Rate)	2021 *n* (% Resistance Rate)	2022 *n* (% Resistance Rate)	2023 *n* (% Resistance Rate)	2024 *n* (% Resistance Rate)
Fluconazole	5 (9.3)	5 (19.2)	10 (23.8)	6 (9.8)	27 (22.3)
Itraconazole	5 (9.3)	6 (23.1)	13 (31.0)	8 (27.6)	23 (20.9)
Voriconazole	ND	ND	ND	1 (3.2)	8 (8.7)
Amphotericin B	0 (0.0)	0 (0.0)	0 (0.0)	2 (3.3)	1 (0.8)
Caspofungin	0 (0.0)	0 (0.0)	0 (0.0)	0 (0.0)	1 (0.8)

Abbreviations: ND: no data available.

**Table 3:** NAC species antifungal resistance rates.

NAC Species	Fluconazole *n* (% Resistance Rate)	Itraconazole *n* (% Resistance Rate)	Amphotericin B *n* (% Resistance Rate)	Micafungin *n* (% Resistance Rate)	Caspofungin *n* (% Resistance Rate)	Anidulafungin *n* (% Resistance Rate)
*N. glabratus*	139 (100)	139 (100)	19 (13.7)	0 (0.0)	19 (13.8)	0 (0.0)
*P. kudriavzevii*	40 (100)	40 (100)	5 (13.2)	ND	ND	ND
*C. parapsilosis*	1 (3.7)	0 (0.0)	0 (0.0)	0 (0.0)	0 (0.0)	0 (0.0)
*C. tropicalis*	2 (12.5)	1 (10.0)	0 (0.0)	ND	0 (0.0)	0 (0.0)

Abbreviations: ND: No data available, *N. glabratus*: *Nakaseomyces glabratus*, *P. kudriavzevii*: *Pichia kudriavzevii*, *C. parapsilosis*: *Candida parapsilosis*, and *C. tropicalis*: *Candida tropicalis*.

## Data Availability

The data presented in this study are available on request from the corresponding author.

## Supplementary Materials

The following supporting information can be downloaded at: https://www.mdpi.com/article/10.3390/pathogens15050538/s1, Table S1: Pathogen distribution across age groups.

## Abbreviations

The following abbreviations are used in this manuscript:

VVC	Vulvovaginal candidosis
NAC	Non-*albicans Candida*
OTC	Over-the-counter
*C. albicans*	*Candida albicans*
*N. glabratus*	*Nakaseomyces glabratus*
*P. kudriavzevii*	*Pichia kudriavzevii*
*C. tropicalis*	*Candida tropicalis*
*C. parapsilosis*	*Candida parapsilosis*
ND	No data available
ReCiDiF	Recurrent *Candida* Infections Treated with Degressive Individualized Doses of Fluconazole
