# Supplementary material for: Evolving Epidemiology and Emerging Antifungal Resistance in Vulvovaginal Candidosis: Evidence from a Five-Year Survey
Source: Pathogens. 2026 May 16;15(5):538. doi: 10.3390/pathogens15050538 (PMC13209537; doi:10.3390/pathogens15050538)
Supplement: Supplementary file 1 [file pathogens-15-00538-s001.zip › pathogens-4290829-supplementary.pdf]

Table S1. Pathogen distribution across age groups.

Age group	*C. albicans* n (% within age group)	*N. glabratus* n (% within age group)	*P.kudriavzevii* n (% within age group)	*C. parapsilosis* n (% within age group)	*C. tropicalis* n (% within age group)
18-29 years	113 (69.9)	27 (17.0)	9 (5.2)	9 (4.6)	7 (3.3)
30-49 years	168 (54.5)	88 (29.3)	28 (9.1)	16 (4.7)	9 (2.4)
50+ years	23 (40.5)	24 (52.4)	3 (4.8)	2 (2.3)	0 (0.0)
Total distribution	304 (57.8)	139 (26.4)	40 (7.6)	27 (5.1)	16 (3.0)
